# Device-measured physical activity data for classification of patients with ventricular arrhythmia events: A pilot investigation

**DOI:** 10.1371/journal.pone.0206153

**Published:** 2018-10-29

**Authors:** Lucas Marzec, Sridharan Raghavan, Farnoush Banaei-Kashani, Seth Creasy, Edward L. Melanson, Leslie Lange, Debashis Ghosh, Michael A. Rosenberg

**Affiliations:** 1 Individualized Data Analysis Organization, Colorado Center for Personalized Medicine, University of Colorado School of Medicine, Aurora, Colorado, United States of America; 2 Division of Cardiology, Kaiser Permanente of Colorado, Lafayette, Colorado, United States of America; 3 Veterans Affairs Eastern Colorado Health Care System, Denver, Colorado, United States of America; 4 Division of General Internal Medicine, University of Colorado School of Medicine, Aurora, Colorado, United States of America; 5 College of Engineering and Applied Science, University of Colorado Denver, Denver, Colorado, United States of America; 6 Division of Endocrinology, Diabetes, Metabolism, University of Colorado School of Medicine, Aurora, Colorado, United States of America; 7 Division of Geriatric Medicine, University of Colorado School of Medicine, Aurora, Colorado, United States of America; 8 Geriatric Research, Education, and Clinical Center, VA Eastern Colorado Health Care System, Denver, Colorado, United States of America; 9 Department of Biostatistics and Informatics, Colorado School of Public Health, Aurora, Colorado, United States of America; 10 Division of Cardiac Electrophysiology, University of Colorado School of Medicine, Aurora, Colorado, United States of America; Kaplan Medical Center, ISRAEL

## Abstract

Low levels of physical activity are associated with increased mortality risk, especially in cardiac patients, but most studies are based on self-report. Cardiac implantable electronic devices (CIEDs) offer an opportunity to collect data for longer periods of time. However, there is limited agreement on the best approaches for quantification of activity measures due to the time series nature of the data. We examined physical activity time series data from 235 subjects with CIEDs and at least 365 days of uninterrupted measures. Summary statistics for raw daily physical activity (minutes/day), including statistical moments (e.g., mean, standard deviation, skewness, kurtosis), time series regression coefficients, frequency domain components, and forecasted predicted values, were calculated for each individual, and used to predict occurrence of ventricular tachycardia (VT) events as recorded by the device. In unsupervised analyses using principal component analysis, we found that while certain features tended to cluster near each other, most provided a reasonable spread across activity space without a large degree of redundancy. In supervised analyses, we found several features that were associated with the outcome (P < 0.05) in univariable and multivariable approaches, but few were consistent across models. Using a machine-learning approach in which the data was split into training and testing sets, and models ranging in complexity from simple univariable logistic regression to ensemble decision trees were fit, there was no improvement in classification of risk over naïve methods for any approach. Although standard approaches identified summary features of physical activity data that were correlated with risk of VT, machine-learning approaches found that none of these features provided an improvement in classification. Future studies are needed to explore and validate methods for feature extraction and machine learning in classification of VT risk based on device-measured activity.

## Background

Implantation of cardiac implantable electrical devices (CIEDs), which include pacemakers and implantable cardioverter-defibrillators (ICDs), has increased dramatically in the past few decades[1]. As the overall functionality of these devices has improved, so has potential for use of data collected by the device in management of patients. In addition to providing treatment through pacemaker and defibrillator functions, CIEDs are capable of collecting a wide range of data parameters on the individuals in whom they are implanted. Among the standard types of information stored and tracked on modern CIEDs includes information about daily physical activity measured by embedded accelerometers, as well as biometric lung impedance monitors to measure breathing rate. This data has been used in CIEDs to moderate pacing to activity level (so-called ‘rate-responsive pacing’) for over 20 years, and have been validated against clinical measurements and external monitors by each of the major manufacturers[2–6]. This information can be stored for customizable durations within the device, as well as uploaded to remote monitoring systems, and thus creates an opportunity to measure with greater precision the daily activities of patients.

One of the challenges of applying physical activity monitor data to clinical outcomes is that there is no clear agreement on how best to model the high-density, time series data that is collected[7–10]. Specific to CIED data, investigators have applied broad summary statistics to these activity time series[11–13], although these methods, which shrink a yearly time series of information down to a single parameter, such as average or last value, lose large amounts of information. Other approaches have modeled data in the frequency domain, which can provide some additional information about long-term seasonality[14], but there remains no clear consensus on how best to model activity data. Nonetheless, investigators are increasingly recognizing that longitudinal measures from physical activity monitors can provide additional predictiveness over simple summary or cross-sectional data from clinic visits[15, 16].

The statistical technique of determining the optimal representation of data prior to use in modeling is called feature engineering or feature extraction[17–19], and is well-known in the machine-learning literature[20]. There are several approaches that investigators have applied to extract features from a time series in order to use the data to model a given outcome. While some approaches, such as using frequency-domain analysis[9, 21] or B-splines[22, 23], have been successful in situations where a given timeframe is meaningful, in others, such as comparing physical activity trends across a population, they do not seem to have as much relevance.

In this investigation, we explore physical activity time series data from patients in whom CIEDs have been implanted using various feature extraction methods, and compare information collected and summarized using unsupervised methods, as well as supervised methods for predicting risk of ventricular tachycardia.

## Methods

### Device data

Physical activity data was available for 355 individuals with Boston Scientific cardiac implantable electronic devices (CIEDs) followed through the Latitude remote monitoring system of the University of Colorado Hospital. The types of CIEDs from which data was collected include single-chamber and dual-chamber permanent pacemakers (PPM) and implantable cardioverter-defibrillators (ICDs), as well as biventricular pacers (also called cardiac resynchronization therapy (CRT) devices) with pacemaker only function (CRT-P) and defibrillator function (CRT-D) as well. For this analysis, we only analyzed data from 235 subjects in whom an entire year of activity data was available. Data was de-identified prior to analysis to remove patient information other than what was collected on the device, and to randomly change dates to maintain privacy, while preserving time-order in such a way that each patient is assigned a new time zero. We opted to examine the outcome of any ventricular tachycardia (VT) events identified by the device (both treated and monitored) as this was the most consistently available outcome unrelated to activity collected by CIEDs for clinical purposes. A subject is defined as having a VT episode if the ICD, which uses a built-in algorithm based on rate, morphology, onset, and atrial-ventricular relationship if an atrial lead is present (dual-chamber ICD), has identified an event as having occurred within the 6-month data collection period. These events can be divided into categories of VT or ventricular fibrillation (VF) by the device based primarily on the rate (VF is faster than VT), but for the purposes of this study, we have included both categories as VT. In general, ICDs do not specifically adjudicate a VT episode as monomorphic or polymorphic, and we were unable to make this determination from the database for this study. Subjects with PPMs implanted were assumed to not have any VT events during the period of study, but are included to improve power of this study based on the assumption that a clinical VT event in these subjects would prompt upgrade to an ICD from a PPM. Unlike ICDs, PPMs do not have built-in algorithms to discriminate VT from high ventricular rates as might be present with supraventricular tachycardia or atrial fibrillation with rapid ventricular rate, and for that reason were excluded. All VT events, including monomorphic and polymorphic that met criteria for VT were included. Nonsustained episodes of VT were excluded. No additional clinical information for patients, including indication for CIED placement, cardiac history, or cardiac function, was available for analysis. The study protocol was approved by the University of Colorado Multiple Institutional Review Board.

### Activity time series feature extraction

Activity was measured in minutes per day. For each subject, the mean, standard deviation, kurtosis, skew, minimum and maximum minutes of activity per day was calculated (See source code in Supplemental Material). A linear model was fit to identify the long-term trend, and the slope and intercept were also stored. To capture autocorrelation structure, the autocorrelation function (ACF) and partial autocorrelation function (PACF) were collected for lags of 1, 2, 3, 7, and 14 days. After detrending by subtracting the slope, a fast Fourier transform was applied to each time series and the period corresponding to the peak of the spectrum was collected for each subject. To predict future activity measures at 7, 14, 30, 60, and 90 days, a seasonal autoregressive integrated moving average (ARIMA) (1, 0, 1)(1, 0, 1)_7_ model was fit to each time series. The coefficients for each subjects’ model (Seasonal AR1, seasonal MA1, AR1, MA1) were also stored for analysis.

### Analysis

**Fig 1:**
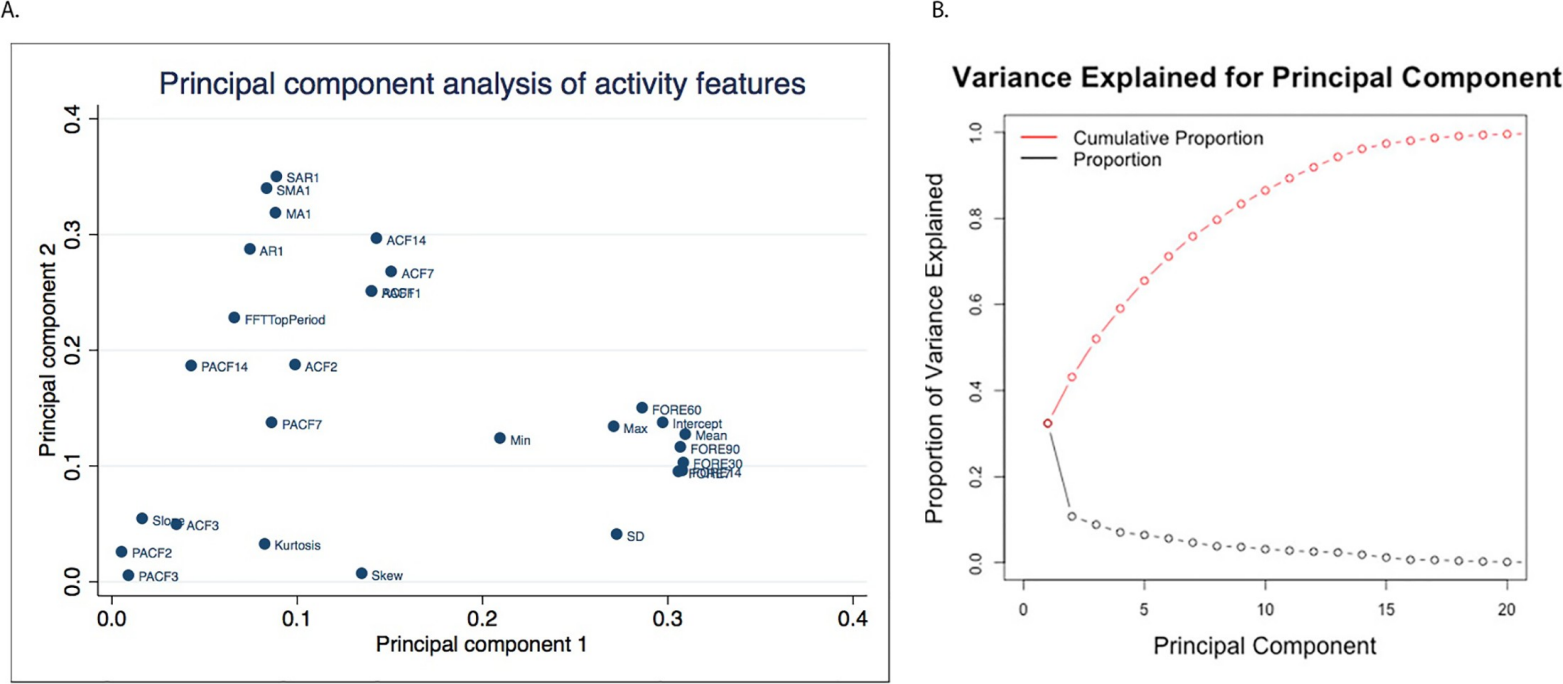
A. First two principal components of activity features. B. Cumulative variance explained by principal components.

All analyses were conducted using R version 3.2.2 (8/14/2015), on RStudio (version 1.0.136). Fig 1A and t-test for proportions of VT events (*prtesti*) were conducted using Stata IC, version 15.0 (Stata, Inc., College Station, TX, USA). Unsupervised analysis of features was performed using *pr*.*comp*::*stats*, with scaling. Univariate supervised analysis, and comparison between types of devices, was performed using a Student’s t test for each feature, grouped by the presence of absence of a ventricular tachycardia event during the study period. Multivariate logistic regression was performed with all features initially, followed by logistic regression with regularization using lasso, ridge, and elastic net regression. Decision tree analysis was performed using *randomForest*::*randomForest*, with boot-strap aggregation and random forest (sampled randomly by 6 features per tree). Boosted decision trees (*gbm*:: *gbm*) were created from 5000 trees at an interaction depth of 4. K-nearest neighbors was performed using k = 1 through k = 10, although only these specific values are reported. Support vector machine analysis was performed using linear (support vector classifier) and radial kernels, with hyper-parameters selected using the *tune*::*e1071* function. Hyper-parameters for all other models were selected 5-fold cross-validation and grid search. Feature importance analysis was performed using *varImpPlot*::*randomForest* after Random Forest analysis, which calculates feature importance using two parameters: the mean decrease in accuracy in predictions on out of bag samples (after that variable is excluded from the model), and a measure of the total decrease in node impurity (*mean decrease in Gini index*) that results from splits over that variable, averaged across all trees[20]. Unless otherwise stated, significance was determined at a level of p < 0.05. R code for feature extraction and analysis of de-identified data is included in Supporting information (S1 File).

## Results

Table 1 shows the types of devices from which activity data was collected. Patients with dual chamber implantable cardioverter-defibrillators (ICDs) had more ventricular tachycardia (VT) episodes during the one-year of monitoring than patients with single chamber ICD (35±7% vs. 18±4%, p < 0.05), with no difference between the number of VT episodes in patients with a cardiac resynchronization device (CRT-D) and other groups. Patients with dual-chamber ICDs were more active than those with single-chamber ICDs, although the difference was not statistically significant (S1 Table; p = 0.17) See Fig A from S1 Fig and Fig B from S1 Fig for examples of activity time series, with summary measures, for representative patients with and without VT events. Table 2 shows the population average values for each feature extracted from the activity time series for each patient. For the top period obtained using frequency domain approaches, the mode is displayed in days, corresponding to close to a weekly (~7 day) cyclical period for most subjects. Noteworthy is that the average linear trend across subjects was slightly negative over time, although forecasted future physical activity was not substantially different from the long-term average (mean), which was also noted from unsupervised analyses (see below).

**Table 1:** Device types and episodes.

Type	Number (%)	Episodes (%)
ICD—Single chamber	88 (37%)	16 (18%)
ICD—Dual chamber	46 (20%)	16 (35%)
CRT-D	59 (25%)	17 (29%)
Pacemaker—Single chamber	6 (3%)	-
Pacemaker—Dual chamber	30 (13%)	-
CRT-P	6 (3%)	-

ICD = Implantable cardioverter-defibrillator, CRT-D = Cardiac resynchronization device-defibrillator, CRT-P = Cardiac resynchronization device-pacemaker

**Table 2:** Physical activity summary information about for each patient (N = 235).

**Moments**		
	Mean	124.0 ± 61.5
	SD	40.3 ± 19.3
	Skew	0.74 ± 0.80
	Kurtosis	1.8 ± 6.1
	Max	277.7 ± 112.4
	Min	34.6 ± 28.3
**Linear Model**		
	Slope	-0.04 ± 0.09
	Intercept	130.6 ± 63.2
**Autocorrelation**		
	ACF1	-0.43 ± 0.09
	ACF2	-0.03 ± 0.10
	ACF7	0.10 ± 0.14
	ACF14	0.11 ± 0.13
	PACF1	-0.43 ± 0.09
	PACF2	-0.27 ± 0.07
	PACF7	-0.06 ± 0.06
	PACF14	-0.03 ± 0.06
**Forecasts**		
	7-day	122.3 ± 67.6
	30-day	123.4 ± 65.3
	60-day	121.1 ± 59.0
	90-day	123.6 ± 65.9
**Frequency**		
	Top Period	6.9

All values except Top Period are mean±standard deviation of physical activity, across all patients. Daily physical activity is measured in minutes/day. Forecasts obtained based on autoregressive integrated moving average (ARIMA) (1, 0, 1) models. Top Period is the mode (in days) across patients, obtained from fast Fourier transform for activity, and corresponds to the highest peak of the frequency plot for each patient. See Methods for details.

A from Fig 1 shows the first and second principal components obtained from principal component analysis (PCA) applied to the features extracted from the activity time series. All 4 time forecasts (i.e., 7-, 30-, 60- and 90-day), mean, and intercept features form the tightest cluster, indicating that they provide similar information regarding daily activity, which was also evident from their similar mean values across the population as a whole (Table 2). B from Fig 1 shows the individual and cumulative variance explained by the principal components, in which these first two components (A from Fig 1) explain ~45% of total variation in physical activity.

**Fig 2:**
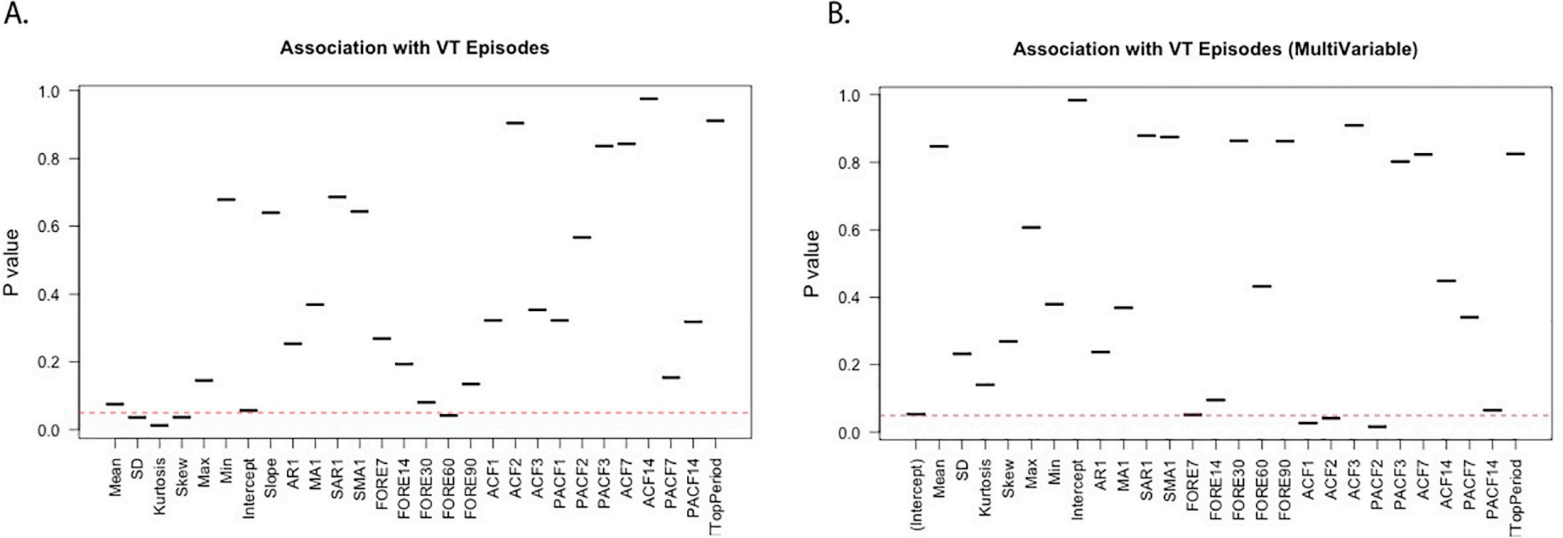
**A. Univariate association with VT episodes (t-test). B. Multivariable logistic regression p-values for association with VT episodes**. Dashed red line: p = 0.05.

There were 49 (20.8%) subjects with at least one ventricular tachycardia (VT) episode during the year of data collection. To examine univariable effect on predicting episodes, we performed a Student’s t test for each separate feature (A from Fig 2). Standard deviation, kurtosis, skew, and 60-day forecast were all associated with VT episodes at p < 0.05. S2 Table shows the direction of effect for each feature, with subjects with VT episodes having an increased standard deviation (45.0 vs. 39.2 min/day, p = 0.036), lower kurtosis (0.76 vs. 2.12, p = 0.013), less skew (0.59 vs. 0.78, p = 0.037), and a higher 60-day forecasted physical activity (135.3 vs. 117.3 min/day, p = 0.042). Neither trend (i.e., slope) nor mean activity, which had been previously associated with mortality[12, 13], was significantly associated with VT risk. In a multivariable logistic regression with all features included (B from Fig 2), the ACF1, ACF2, and PACF2 were all associated with VT episodes at p < 0.05, with increased ACF1 and ACF2 being associated with decreased risk of a VT episode and increased PACF2 being associated with an increased risk of a VT episode.

**Fig 3:**
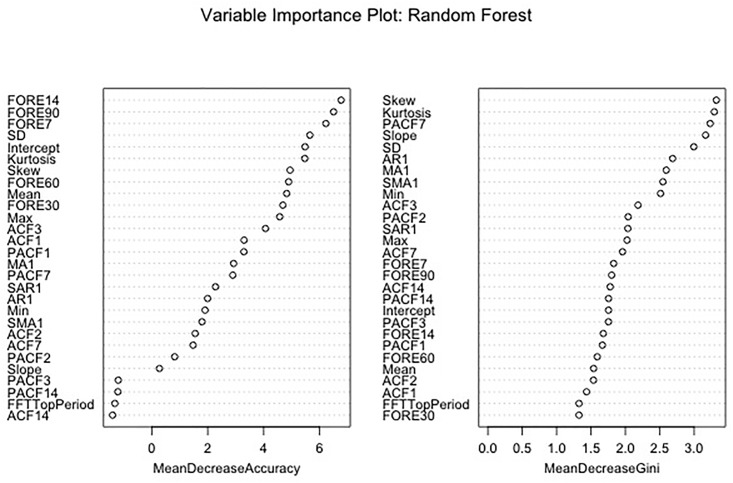
Variable importance plot. From random forest model.

To provide additional assessment of relative feature importance, we examined feature importance analysis after several machine-learning approaches, including random forests (Fig 3) and boosted and bagged tree models (A and B from S2 Fig). Noteworthy is that all models generally identified different features in terms of greater importance in predicting VT episodes, some of which, such as skewness, overlapped with logistic regression models (above), but most of which were unique to only one model.

To explicitly examine the predictive ability of models, including those above, to use physical activity data to predict VT episodes, we split the data into training (80%) and testing sets (20%). Of note, the percentage of VT episodes in the testing set was 25.5% (12 episodes in 47 subjects), and so a naïve classifier that always predicted no episode would be accurate 74.5% of the time (see Table 3). Neither univariate logistic regression models based on standard deviation, skew, kurtosis, or 60-day forecast, nor multivariable logistic regression models provided any additional predictive power over a naïve classifier. After performing a number of analyses using various machine-learning approaches (Table 3), there was no clear improvement in classification of VT episodes using any particular approach, with most performing no better, or even worse, than random chance (AUC = 0.5).

**Table 3:** Predictive accuracy of different models for VT episodes.

	Accuracy	AUC	F1 score
Naïve	74.5%	0.50	NA
UV Logistic Regression	74.5%	0.50	NA
MV Logistic Regression	70.2%	0.61	0.417
Penalized Logistic Regression	74.5%	0.50	NA
Bagged Decision Tree	74.5%	0.55	0.250
Random Forest	76.6%	0.57	0.267
Boosted Decision Tree	70.2%	0.50	0.125
KNN (k = 1)	55.3%	0.43	0.16
KNN (k = 10)	72.3%	0.49	0.00
SVC	74.5%	0.50	NA
SVM	74.5%	0.50	NA

Note: Penalized Logistic Regression includes lasso, ridge, and elastic net regression models (result was same across models). UV = Univariable (Standard deviation, skew, kurtosis, and 60-day forecast, separately), MV = Multivariable, KNN = K-nearest neighbors classifier, SVC = Support vector classifier, SVM = Support vector machine, AUC = Area under receiver operator curve. F1 score is the harmonic average of precision and recall (range 0–1).

## Discussion

In this pilot study using physical activity time series data obtained from CIEDs, we found that an approach that extracts moments (mean, standard deviation, etc.), frequency domain measures, and patterns of autocorrelation, but not forecasted values, provides reasonably orthogonal information when examined using unsupervised approaches. This finding is important, as it indicates that such a method does capture additional information beyond standard measures such as mean, last value, or standard deviation, that have been used in predictive models previously. Perhaps more importantly, however, was our finding that although standard univariable approaches identified several possible features extracted from a physical activity time series that were associated with VT episodes at p < 0.05, there was no confirmation of these associations using machine-learning approaches and data splitting for validation. This latter finding is important because it highlights the importance of using prediction of new data as the ultimate measure by which results from data mining studies such as this should be compared, rather than fit to a full dataset as is often performed in practice.

The expansive use of wearable physical activity and heart rate monitors is increasing the opportunity to collect additional patient lifestyle information beyond what can be measured in a clinic setting. In contrast to most wearable physical activity monitors, which suffer from limited long-term compliance by users[24–26], CIEDs are capable of daily, unbiased collection of data throughout the course of their implantation, which is generally on the order of years, and with re-implantation of a new device for most patients. However, there remains a large void in terms of how the information collected by a CIED about daily physical activity could be used by providers for disease prediction. In this respect, CIEDs present a potential opportunity whereby investigators might examine physical activity data collected by the device and compare it with information about cardiac arrhythmia events as we have done here in this study, or other external information available through medical record examination. To date, several investigators have started to utilize this potential fund of information, albeit through use of very simple summary statistics. Kramer et al., examined the average daily physical activity trend at two time points 6 months apart[13] and as a linear time-varying covariate[12] and identified a correlation between decreased physical activity trend and mortality, although such an approach failed to capture seasonality, cyclical trends, or overall patterns in variability, all of which have been associated with outcomes in studies of wearable (i.e., non-implanted) physical activity monitor data[14, 27–32]. With innovations in technology, such as development of implantable cardiac event monitors as small as a pen cap[33–35], it is likely that the numbers and complexity of analyses using this type of data will only grow.

Time series data presents a unique challenge in predictive modeling, in that it can contain large amounts of information about individual patterns of behavior or biological measures, and yet efforts to use this information to its fullest extent tend to lead to difficult clinical interpretation. For example, while interpretation of the mean activity over a period of time, or the standard deviation/variance of activity, can be linked to a specific characteristic of lifestyle or behavior, it is less clear how one might interpret the kurtosis or skewness of daily physical activity, much less an autocorrelation function. In many ways, this problem highlights the challenges of machine learning as a whole, where ‘black-box’ approaches might identify a given prediction for an individual, but without the ability to translate the model parameters in a clinically meaningful way, it is difficult to expect patients or providers to apply these models[36]. This problem becomes even more evident with development of deep learning models for classification of time series, such as long short-term memory recurrent neural networks[37]. Clearly more work is needed, perhaps targeted to outcomes more likely to be associated with daily physical activity than ventricular tachycardia, such as weight loss[38], frailty[39], or mortality[12].

### Strengths

Among the strengths of this study was our finding that although several features derived from physical activity time series data were themselves ‘significant’ at p < 0.05 in standard univariate or multivariate models, no model improved prediction of events over random chance when examined within the framework of a machine-learning approach. This characteristic is important as it highlights how machine learning maintains focus on overall model prediction, rather than identification of associated features, or risk factors, as has become standard practice in many epidemiology studies[36, 40]. While different machine-learning approaches provide different strengths and weaknesses, often described as the bias-variance tradeoff[20, 41], the overall goal of predicting outcomes in held-out, or testing, data provides an important guide in model selection and interpretation. One might conclude from the univariate analysis that skewness in daily physical activity over a year could have some impact on risk of events, perhaps through behavior that leads to extreme deviations over the course of the year. Such interpretation has been applied to continuous glucose monitoring data[42], and could be applied in our study were it not for the lack of validation achieved with a machine-learning approach.

### Weaknesses/Limitations

Although we achieved the overall goal of this pilot study, aimed to examine methods of feature extraction from a physical activity time series for predictive purposes, there were several key limitations. For one, we did not model the temporal relationships between the outcome and physical activity, which others have shown to provide evidence of an association for other conditions such as atrial fibrillation[14]. We decided *a priori* that the addition of temporal parameters might decrease the power of this already small pilot study, but we acknowledge that for these models to be clinically applicable, incorporation of temporality is a key requisite. Another limitation was that we made the assumption that subjects with pacemakers did not have any VT episodes during the one year of follow-up. Because of the de-identified nature of the dataset, we could not obtain outside information to confirm the absence of any VT, although it is not an unreasonable assumption, as we suspect that any clinical VT could have led to device upgrade if it occurred in a pacemaker patient, and that this subject would not have been able to complete an entire year of data collection on the same device if such an upgrade took place. This limitation also extends to the indication for implantation of the CIED, which was also not available as part of the deidentified dataset. This information could have relevance as in certain ventricular arrhythmia syndromes, such as long QT syndrome, Type 1, and catecholaminergic polymorphic VT syndrome, increased physical activity can promote the arrhythmias, while in patients with underlying congestive heart failure, a clinical exacerbation that caused a decrease in physical activity could also result in increased risk of VT. Further work with identifiable datasets is needed to explore these disease-specific hypotheses.

### Conclusion

We found that feature extraction from daily physical activity time series data from a CIED provides reasonable coverage of feature space, and that machine-learning approaches, particularly validation sets, are requisite for investigations using this approach to clinical prediction. Future studies including large datasets and separately adjudicated clinical outcomes will be needed to identify additional application of CIED physical activity data in the clinical setting.

## Supporting information

S1 Fig Representative activity time series and summary measures for 3 subjects without (Fig A) and with (Fig B) VT events. Left, activity time series with long-term average (red), linear trend (blue), and 60-day forecast (green) with errors. Linear trend obtained from slope of linear model of daily activity~time. Forecast obtained from seasonal ARIMA(1,0,1)_7_ model, as described in Methods. Right, frequency domain tracing from fast Fourier transform for each activity time series. Provided are top 5 periods (red dashed lines) based on peaks [Note: Analysis for this study evaluated the top frequency/period for each subject].
(TIF)

S2 Fig **A. Variable Influence Plot from Boosted Tree model**. Obtained using out-of-bag estimate of relative influence for each feature. SD = Standard deviation, AR = Coefficient from autoregressive-1 term, MA1 = Coefficient from moving average-1 term, SAR1 = Coefficient from seasonal (7 day) autoregressive-1 term, SMA1 = Coefficient from seasonal (7-day) moving average-1 term, ACF1-14 = Autocorrelation function, lags 1–14, PACF1-14 = Partial autocorrelation function, lags 1–14. ARIMA model [1, 0, 1][1, 0, 1]_7_ used for coefficients and forecasts. **B. Variable Importance Plots from Bagged Tree models**. Left, mean decrease in model accuracy using out-of-bag exclusion. Right, mean decrease in Gini Index based on total decrease in node impurity with out-of-bag exclusion. SD = Standard deviation, AR = Coefficient from autoregressive-1 term, MA1 = Coefficient from moving average-1 term, SAR1 = Coefficient from seasonal (7 day) autoregressive-1 term, SMA1 = Coefficient from seasonal (7-day) moving average-1 term, ACF1-14 = Autocorrelation function, lags 1–14, PACF1-14 = Partial autocorrelation function, lags 1–14. ARIMA model [1, 0, 1][1, 0, 1]_7_ used for coefficients and forecasts. See Methods for details.
(TIF)

S1 Table Average activity by device type. CRT-D = Cardiac resynchronization therapy (Biventricular) with defibrillator; CRT-P = Cardiac resynchronization therapy (Biventricular) with pacemaker only; DC-ICD = Dual-chamber implantable cardioverter-defibrillator; DC-PPM = Dual-chamber pacemaker; SC-ICD = Single-chamber implantable cardioverter-defibrillator; SC-PPM = Single-chamber pacemaker.
(DOCX)

S2 Table Estimates from univariate feature analysis. SD = Standard deviation, AR = Coefficient from autoregressive-1 term, MA1 = Coefficient from moving average-1 term, SAR1 = Coefficient from seasonal (7 day) autoregressive-1 term, SMA1 = Coefficient from seasonal (7-day) moving average-1 term, ACF1-14 = Autocorrelation function, lags 1–14, PACF1-14 = Partial autocorrelation function, lags 1–14. ARIMA model [1, 0, 1][1, 0, 1]_7_ used for coefficients and forecasts.
(DOCX)

S1 File R-code for analysis. (R)
