# Supplementary figures and images for: Device-measured physical activity data for classification of patients with ventricular arrhythmia events: A pilot investigation
Source: PLoS One. 2018 Oct 29;13(10):e0206153. doi: 10.1371/journal.pone.0206153 (PMC6205644; doi:10.1371/journal.pone.0206153)

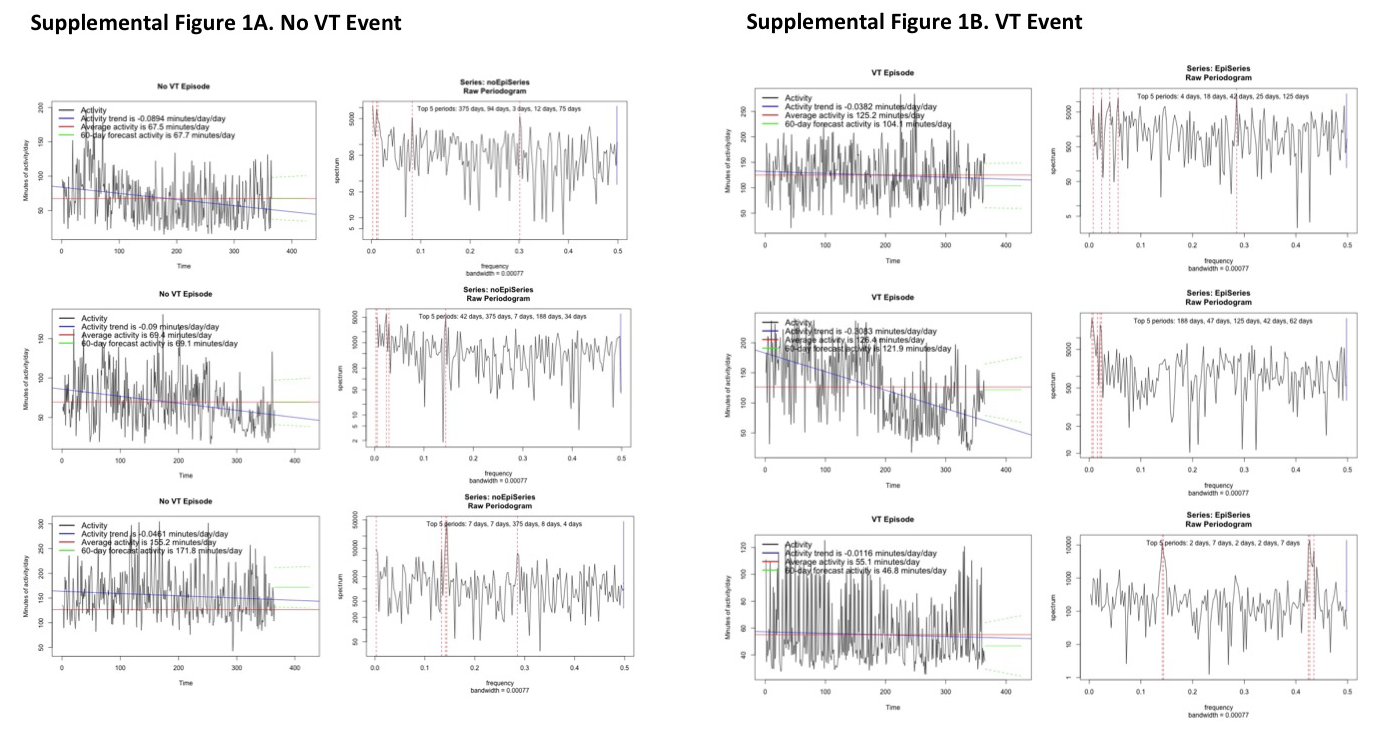

Supplement: S1 Fig — Left, activity time series with long-term average (red), linear trend (blue), and 60-day forecast (green) with errors. Linear trend obtained from slope of linear model of daily activity~time. Forecast obtained from seasonal ARIMA(1,0,1)7 model, as described in Methods. Right, frequency domain tracing from fast Fourier transform for each activity time series. Provided are top 5 periods (red dashed lines) based on peaks [Note: Analysis for this study evaluated the top frequency/period for each subject]. (TIF) [file pone.0206153.s001.tif]

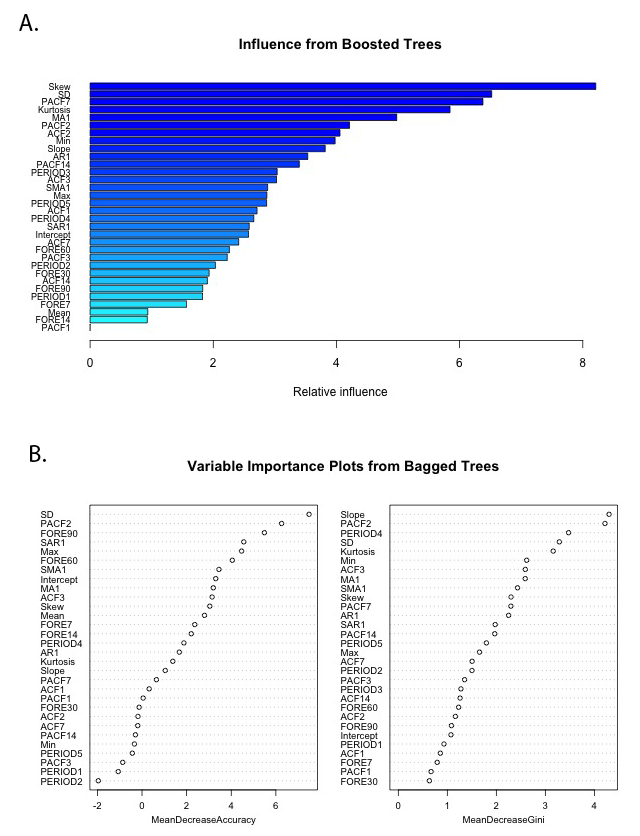

Supplement: S2 Fig — A. Variable Influence Plot from Boosted Tree model. Obtained using out-of-bag estimate of relative influence for each feature. SD = Standard deviation, AR = Coefficient from autoregressive-1 term, MA1 = Coefficient from moving average-1 term, SAR1 = Coefficient from seasonal (7 day) autoregressive-1 term, SMA1 = Coefficient from seasonal (7-day) moving average-1 term, ACF1-14 = Autocorrelation function, lags 1–14, PACF1-14 = Partial autocorrelation function, lags 1–14. ARIMA model [1, 0, 1][1, 0, 1]7 used for coefficients and forecasts. B. Variable Importance Plots from Bagged Tree models. Left, mean decrease in model accuracy using out-of-bag exclusion. Right, mean decrease in Gini Index based on total decrease in node impurity with out-of-bag exclusion. SD = Standard deviation, AR = Coefficient from autoregressive-1 term, MA1 = Coefficient from moving average-1 term, SAR1 = Coefficient from seasonal (7 day) autoregressive-1 term, SMA1 = Coefficient from seasonal (7-day) moving average-1 term, ACF1-14 = Autocorrelation function, lags 1–14, PACF1-14 = Partial autocorrelation function, lags 1–14. ARIMA model [1, 0, 1][1, 0, 1]7 used for coefficients and forecasts. See Methods for details. (TIF) [file pone.0206153.s002.tif]
