# Supplementary material for: Device-measured physical activity data for classification of patients with ventricular arrhythmia events: A pilot investigation
Source: PLoS One. 2018 Oct 29;13(10):e0206153. doi: 10.1371/journal.pone.0206153 (PMC6205644; doi:10.1371/journal.pone.0206153)
Supplement: S1 Table — CRT-D = Cardiac resynchronization therapy (Biventricular) with defibrillator; CRT-P = Cardiac resynchronization therapy (Biventricular) with pacemaker only; DC-ICD = Dual-chamber implantable cardioverter-defibrillator; DC-PPM = Dual-chamber pacemaker; SC-ICD = Single-chamber implantable cardioverter-defibrillator; SC-PPM = Single-chamber pacemaker. (DOCX) [file pone.0206153.s003.docx]

**Supplemental Table 1. Average activity by device type**

| **Device** | **Mean Activity (min/day)** | **Standard Deviation Activity (min/day)** | **Number of devices** |
| --- | --- | --- | --- |
| CRT-D | 109.27 | 57.27 | 59 |
| CRT-P | 70.96 | 40.19 | 6 |
| DC-ICD | 144.81 | 56.00 | 46 |
| DC-PPM | 105.99 | 56.56 | 30 |
| SC-ICD | 130.52 | 57.39 | 88 |
| SC-PPM | 156.82 | 140.01 | 6 |

CRT-D = Cardiac resynchronization therapy (Biventricular) with defibrillator; CRT-P = Cardiac resynchronization therapy (Biventricular) with pacemaker only; DC-ICD = Dual-chamber implantable cardioverter-defibrillator; DC-PPM = Dual-chamber pacemaker; SC-ICD = Single-chamber implantable cardioverter-defibrillator; SC-PPM = Single-chamber pacemaker;
