# Supplementary material for: Device-measured physical activity data for classification of patients with ventricular arrhythmia events: A pilot investigation
Source: PLoS One. 2018 Oct 29;13(10):e0206153. doi: 10.1371/journal.pone.0206153 (PMC6205644; doi:10.1371/journal.pone.0206153)
Supplement: S2 Table — SD = Standard deviation, AR = Coefficient from autoregressive-1 term, MA1 = Coefficient from moving average-1 term, SAR1 = Coefficient from seasonal (7 day) autoregressive-1 term, SMA1 = Coefficient from seasonal (7-day) moving average-1 term, ACF1-14 = Autocorrelation function, lags 1–14, PACF1-14 = Partial autocorrelation function, lags 1–14. ARIMA model [1, 0, 1][1, 0, 1]7 used for coefficients and forecasts. (DOCX) [file pone.0206153.s004.docx]

**Supplemental Table 2. Estimates from Univariate Feature Analysis**

| Feature | Mean (without episode) | Mean (with episode) | Estimate 95% Confidence interval (lower, upper) | p value |
| --- | --- | --- | --- | --- |
| Mean | 120.6 | 136.8 | -34.0, 1.7 | 0.075 |
| SD | 39.2 | 45.0 | -11.1, -0.4 | 0.036 |
| Kurtosis | 2.13 | 0.76 | 0.29, 2.43 | 0.013 |
| Skewness | 0.78 | 0.59 | 0.01, 0.37 | 0.037 |
| Maximum | 272.7 | 296.7 | -56.7, 8.5 | 0.145 |
| Minimum | 34.2 | 36.1 | -10.9, 7.1 | 0.679 |
| Intercept | 126.9 | 144.5 | -35.7, 0.5 | 0.057 |
| Slope | -0.03 | -0.04 | -0.03, 0.04 | 0.640 |
| AR1 | 0.69 | 0.60 | -0.06, 0.23 | 0.254 |
| MA1 | -0.49 | -0.43 | -0.20, 0.07 | 0.370 |
| SAR1 | 0.61 | 0.57 | -0.15, 0.23 | 0.687 |
| SMA1 | -0.55 | -0.50 | -0.23, 0.14 | 0.644 |
| 7-day Forecast | 120.1 | 130.5 | -29.1, 8.2 | 0.269 |
| 14-day Forecast | 120.0 | 132.3 | -30.9, 6.3 | 0.193 |
| 30-day Forecast | 120.0 | 136.4 | -34.9, 2.0 | 0.081 |
| 60-day Forecast | 117.3 | 135.3 | -35.2, -0.7 | 0.042 |
| 90-day Forecast | 120.7 | 134.6 | -32.1, 4.4 | 0.135 |
| ACF1 | -0.43 | -0.44 | -0.01, 0.04 | 0.323 |
| ACF2 | -0.03 | -0.03 | -0.03, 0.03 | 0.904 |
| ACF3 | -0.03 | -0.02 | -0.03, 0.01 | 0.354 |
| ACF7 | 0.11 | 0.10 | -0.04, 0.05 | 0.843 |
| ACF14 | 0.11 | 0.11 | -0.04, 0.04 | 0.976 |
| PACF1 | -0.43 | -0.44 | -0.01, 0.04 | 0.323 |
| PACF2 | -0.27 | -0.28 | -0.01, 0.02 | 0.567 |
| PACF3 | -0.20 | -0.20 | -0.02, 0.02 | 0.837 |
| PACF7 | -0.06 | -0.07 | -0.01, 0.03 | 0.154 |
| PACF14 | -0.03 | -0.02 | -0.03, 0.01 | 0.318 |
| Top Period | 108.3 | 110.6 | -43.5, 38.8 | 0.911 |

SD = Standard deviation, AR = Coefficient from autoregressive-1 term, MA1 = Coefficient from moving average-1 term, SAR1 = Coefficient from seasonal (7 day) autoregressive-1 term, SMA1 = Coefficient from seasonal (7-day) moving average-1 term, ACF1-14 = Autocorrelation function, lags 1-14, PACF1-14 = Partial autocorrelation function, lags 1-14. ARIMA model [1, 0, 1][1, 0, 1]_7_ used for coefficients and forecasts
